# A potential role for the paraventricular nucleus of the thalamus in mediating individual variation in Pavlovian conditioned responses

**DOI:** 10.3389/fnbeh.2014.00079

**Published:** 2014-03-14

**Authors:** Joshua L. Haight, Shelly B. Flagel

**Affiliations:** ^1^Neuroscience Graduate Program, University of MichiganAnn Arbor, MI, USA; ^2^Department of Psychiatry, University of MichiganAnn Arbor, MI, USA; ^3^Molecular and Behavioral Neuroscience Institute, University of MichiganAnn Arbor, MI, USA

**Keywords:** paraventricular nucleus of the thalamus, sign-tracking, goal-tracking, cue-learning, motivated behavior, addiction, incentive salience, incentive stimuli

## Abstract

There is ample evidence to suggest that the paraventricular nucleus of the thalamus (PVT) mediates cue-reward learning, especially as it relates to drug-seeking behavior. However, its exact role in these complex processes remains unknown. Here we will present and discuss data from our own laboratory which suggests that the PVT plays a role in multiple forms of stimulus-reward learning, and does so via distinct neurobiological systems. Using an animal model that captures individual variation in response to reward-associated cues, we are able to parse the incentive from the predictive properties of reward cues and to elucidate the neural circuitry underlying these different forms of cue-reward learning. When rats are exposed to a classical Pavlovian conditioning paradigm, wherein a cue predicts food reward, some rats, termed sign-trackers, approach and manipulate the cue upon its presentation. This behavior is indicative of attributing incentive salience to the cue. That is, the cue gains excessive control over behavior for sign-trackers. In contrast, other rats, termed goal-trackers, treat the cue as a mere predictor, and upon its presentation go to the location of reward delivery. Based on our own data utilizing this model, we hypothesize that the PVT represents a common node, but differentially regulates the sign- vs. goal-tracking response. We postulate that the PVT regulates sign-tracking behavior, or the attribution of incentive salience, via subcortical, dopamine-dependent mechanisms. In contrast, we propose that goal-tracking behavior, or the attribution of predictive value, is the product of “top-down” glutamatergic processing between the prelimbic cortex (PrL) and the PVT. Together, data from our laboratory and others support a role for the PVT in cue-motivated behaviors and suggest that it may be an important locus within the neural circuitry that goes awry in addiction and related disorders.

## Introduction

Over the past few decades a large quantity of research has focused on elucidating the neurobiological mechanisms that contribute to addiction and related behaviors (for review see: Grace, 2000; Kelley and Berridge, 2002; Lüscher and Malenka, 2011; Everitt and Robbins, 2013; Nestler, 2014). The majority of this work has focused on the classic mesocorticolimbic reward circuitry, but the field is beginning to recognize the importance of structures outside of this system (e.g., Ikemoto, 2010). One such structure is the paraventricular nucleus of the thalamus (PVT), which has recently gained attention for its role in mediating cue-driven behaviors, especially as they relate to drug-seeking behavior and addiction (Martin-Fardon and Boutrel, 2012; James and Dayas, 2013; Browning et al., 2014). Although there is now sufficient evidence implicating the PVT in mediating responses to both food- and drug-associated cues, its exact role in these processes has yet to be discovered.

The PVT is a midline thalamic nucleus located at the interface between the limbic, cortical and motor circuits. The PVT receives a complex set of sub-cortical afferents from areas known to be involved in motivated behavior, including the hypothalamus, hippocampus, amygdala, locus coeruleus, periaqueductal grey, and dorsal raphe (Van der Werf et al., 2002; Vogt et al., 2008; Hsu and Price, 2009; Li and Kirouac, 2012). In addition to these sub-cortical elements, the PVT receives strong innervation from the medial prefrontal cortex, including the prelimbic, infralimbic, cingulate and dorsal peduncular cortices (Li and Kirouac, 2012). The densest set of afferents to the PVT appears to be from the prelimbic cortex (PrL; Li and Kirouac, 2012), an area recently shown to be a critical mediator of drug- and cue-motivated behaviors (Di Pietro et al., 2006; Di Ciano et al., 2007; Rocha and Kalivas, 2010). The efferents from the PVT are primarily glutamatergic, targeting both cortical and subcortical structures including the PrL, nucleus accumbens (NAc) shell and core, amygdala, hippocampus, and hypothalamus (Jones et al., 1989; Su and Bentivoglio, 1990; Van der Werf et al., 2002; Pinto et al., 2003; Li and Kirouac, 2008; Vertes and Hoover, 2008). Thus, the neuroanatomical positioning of the PVT is ideal for integrating information regarding environmental stimuli and internal states and translating it into motivated actions.

The first study to implicate the PVT as a potential mediator of motivated behavior surfaced almost 50 years ago when it was demonstrated that rats will self-stimulate intracranial electrodes placed in or near the PVT (Cooper and Taylor, 1967). These findings were later supported by Clavier and Gerfen (1982), who confirmed that the most consistent patterns of thalamic self-stimulation occurred when electrode placements were close to, or within the midline nuclei, which included the PVT. Since then, numerous studies have supported a role for the PVT in motivated behavior, specifically in response to discrete and contextual cues that have previously been paired with food and drug rewards. Here we review behavioral, pharmacological, and anatomical evidence supporting a role for the PVT in cue-motivated behaviors and, based on our own data, discuss a potential role for this structure in mediating specific aspects of cue-reward learning and Pavlovian conditioned approach behaviors.

## A role for the paraventricular nucleus of the thalamus (PVT) in reward processing and cue-motivated behaviors

More than a decade following the intracranial self-stimulation studies (Cooper and Taylor, 1967; Clavier and Gerfen, 1982), the PVT was shown to play a role in psychoactive drug effects. Systemic administration of amphetamine and 3,4-methylenedioxy-N-methylamphetamine (MDMA) elicits an increase in neuronal activity in the PVT, as measured by *c-fos* (Deutch et al., 1995, 1998; Stephenson et al., 1999). Around this same time, a series of lesion studies sought to examine the role of the PVT in cocaine-induced behavioral sensitization. It was found that lesions of the PVT before (Young and Deutch, 1998), but not after (Pierce et al., 1997), a contextually conditioned regimen of repeated cocaine treatment attenuates the development of behavioral sensitization. These studies were the first to suggest that the PVT was important for the acquisition of the relationship between drugs and conditioned stimuli.

By this time it had been well established that motivated behaviors, such as behavioral sensitization, are regulated by a complex set of cortical, striatal, thalamic and limbic brain areas, known as the “motive circuit” (for review see Pierce and Kalivas, 1997). However, it wasn’t until later that work by Ann Kelley et al. highlighted the PVT as an important component of this circuitry (Kelley et al., 2005a). In Kelley’s model, the PVT is a critical interface between the limbic and motor circuitry, relaying information regarding arousal, environmental cues, energy needs, reward, and circadian rhythms from the hypothalamus to the striatum, including the NAc. Once in the striatum, this information is incorporated with other salient information from the ventral tegmental area (VTA) and prefrontal cortex, among other areas, and integrated with basal-ganglia motor output pathways to influence motivated behaviors. In support of this model, Kelley et al. demonstrated that exposure to a context previously paired with a highly palatable reward (chocolate Ensure) can induce robust cellular activation throughout many areas of the motive circuitry, including prefrontal cortical areas, the amygdala, NAc, and the PVT (Schiltz et al., 2005a, 2007). Interestingly, exposure to a context previously paired with nicotine administration also induces robust cellular activation in these areas (Schiltz et al., 2005b). This similar pattern of neuronal activation in response to both food and drug cues led Kelley et al. to postulate that “addictive drugs induce neuroadaptations in brain circuits normally subserving learning and memory for motivationally salient stimuli” (pg. 12, Kelley et al., 2005b), and the PVT appears to be a critical locus of these circuits.

Recent behavioral studies have built upon the initial studies by Kelley et al. (Kelley et al., 2005b; Schiltz et al., 2005a,b, 2007), further supporting the notion that the PVT is an important mediator of contextual cue-reward associations and addiction-related behaviors (Martin-Fardon and Boutrel, 2012; James and Dayas, 2013). Johnson et al. demonstrated that exposure to a context previously paired with repeated experimenter administered cocaine injections increases levels of *c-fos* in the PVT (Johnson et al., 2010). Moreover, lesions or chemical inactivation of the PVT prevent reinstatement of “beer-seeking” behavior following exposure to the previously alcohol-paired context (Hamlin et al., 2009; Marchant et al., 2010). Additionally, the expression of cocaine-induced conditioned place preference is attenuated following inactivation of the PVT (Browning et al., 2014), further confirming a role for the PVT in contextual cue-reward processes.

Similar findings have been published with discrete reward-paired cues. For example, repeated Pavlovian pairings of a discrete cue light with a water reward results in increased *c-fos* expression in the PVT relative to unpaired controls (Igelstrom et al., 2010). Likewise, exposure to a discrete odor cue previously associated with ethanol availability in a Pavlovian manner increases *c-fos* expression in the PVT (Dayas et al., 2008). *C-fos* is also elevated in the PVT following reinstatement of drug-seeking behaviors after exposure to ethanol- (Wedzony et al., 2003) or cocaine-associated (James et al., 2011) cues. Further, drug-seeking behavior can be disrupted by inactivation of the PVT, as James et al. demonstrated that a direct infusion of tetrodotoxin (a voltage-gated sodium channel antagonist) or the inhibitory peptide cocaine- and amphetamine-regulated transcript (CART) into the PVT is able to attenuate cocaine-primed reinstatement (James et al., 2010). Taken together, these findings demonstrate a role for the PVT in the conditioned-effects of both discrete and contextual reward-associated cues, and drug-seeking behavior.

### PVT-dopamine interactions relevant to reward processing and cue-motivated behaviors

A series of elegant studies have been published that further support a role for the PVT in motivated behaviors via its interactions with the dopamine system. Mesocorticolimbic dopamine transmission has long been known to play a role in cue- and drug-motivated behavior. Exposure to food or drug rewards, as well as reward-paired cues, elicits robust dopamine transmission in the NAc (for review see Baik, 2013). The PVT sends projections to the NAc core and, to a greater extent, the shell (Van der Werf et al., 2002; Li and Kirouac, 2008; Vertes and Hoover, 2008), and many of the these neurons are found in close proximity to tyrosine-hydroxylase positive (i.e., dopaminergic) axons (Pinto et al., 2003). This is one mechanism by which PVT activity can influence dopamine release in the ventral striatum. Another possibility is that the PVT affects accumbens dopamine activity by modulating presynaptic terminals. In support, Parsons et al. (2007) demonstrated that electrical excitation of the PVT elicits dopamine efflux independent of the VTA; and showed that these PVT-evoked responses were attenuated following intra-accumbens infusion of a glutamate receptor antagonist. Thus, glutamate release from PVT terminals appears to presynaptically regulate accumbens dopamine activity (Parsons et al., 2007). It has also been postulated that hypothalamic orexin neurons that project to the PVT are part of the sub-cortical system that drives dopamine levels in the ventral striatum (Kelley et al., 2005a). In support, *in vivo* administration of orexin-a peptide directly into the PVT has been shown to increase dopamine levels in the NAc (Choi et al., 2012). Thus, there are multiple ways in which the PVT can influence dopamine activity in the NAc, and in turn regulate motivated behaviors.

The PVT also receives sub-cortical input from dopaminergic neurons (Lindvall et al., 1984; García-Cabezas et al., 2009). Early biochemical evidence demonstrated that dopamine innervation of the PVT was, at least in part, coming from the VTA cell group in the ventromedial midbrain (Kizer et al., 1976). This was later supported by a retrograde tracing study showing that dopaminergic cells (i.e., tyrosine-hydroxylase positive) in the VTA projected to the PVT (Takada et al., 1990). However, it should be noted that tracing studies from other groups have not identified a circuit between the VTA and PVT (Cornwall and Phillipson, 1988; Chen and Su, 1990; Li and Kirouac, 2012). Alternatively, it has been suggested that dopaminergic innervation of the PVT arises primarily from the A11, A13 and A14 cell groups residing in the hypothalamus and periaqueductal gray (Lindvall et al., 1984). In support, it has since been demonstrated in monkeys that dopaminergic input to midline thalamic nuclei (PVT and centromedial nucleus combined) is coming from these cell groups, with the hypothalamus being the major source of input (Sánchez-González et al., 2005). Retrograde transport from the PVT is evident in these same brain regions in rats (Chen and Su, 1990; Li and Kirouac, 2012), but there are cross-species differences in the pattern and density of dopaminergic innervation of the thalamus (García-Cabezas et al., 2009). Thus, further work is warranted to characterize the sources of dopaminergic input to the rat PVT.

**Figure 1:**
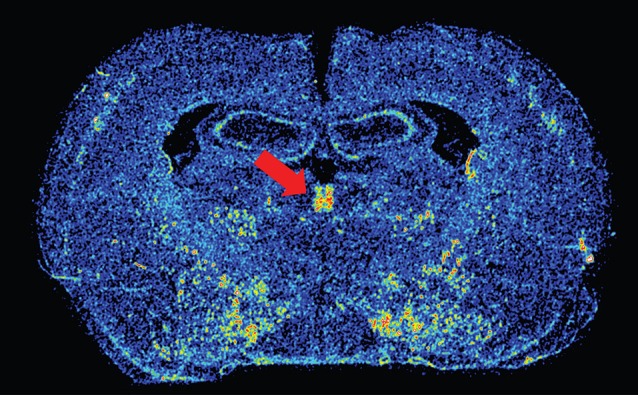
**Image of dopamine D3 receptor mRNA expression.** Color-enhanced *in situ* hybridization image of D3 mRNA in the PVT (red arrow) in a coronal rat brain section. Approximate Bregma level is –2.28 (Paxinos and Watson, 2007).

Dopamine in the PVT presumably acts on dopamine D3 receptors, the primary dopamine receptor in the PVT (Mansour and Watson, 1995). We have recently confirmed the presence of D3 mRNA in the PVT using *in situ* hybridization, and remarkably, D3 expression is restricted to the PVT and not apparent in any of the surrounding thalamic nuclei (Figure 1). While the specific role of D3 activation in the PVT has yet to be examined, recent reports have demonstrated that systemic antagonism of D3 receptors can block both drug- and cue-induced reinstatement of drug-seeking behaviors (Xi et al., 2006; Peng et al., 2009; Khaled et al., 2010; Higley et al., 2011; Rice et al., 2013). Interestingly, unpublished data from our own lab suggests that rats that are more susceptible to both drug- and cue-induced reinstatement have greater D3 mRNA expression in the PVT. Human imaging studies have also associated dopaminergic transmission in the thalamus with addiction-related behavior. Work by Volkow et al. has shown that methylphenidate administration in cocaine abusers leads to increased dopamine levels in the thalamus, which is positively correlated with reports of drug craving (Volkow et al., 1997).The resolution in human imaging studies does not allow one to distinguish the PVT from other thalamic nuclei, but these results are nonetheless interesting and relevant. Taken together, the literature reviewed above led us to postulate that the PVT influences cue- and reward-motivated behaviors by integrating information from sub-cortical systems, such as the orexin and dopamine neurotransmitter systems, and relaying that information to the ventral striatum, where it can impact NAc activity.

## Exploiting individual variation in pavlovian conditioned responses to parse the role of the paraventricular nucleus of the thalamus (PVT) in cue-reward learning

As summarized above, there is now sufficient evidence supporting the involvement of the PVT in motivated behavior and the processing of reward-associated cues. However, it is difficult to draw conclusions about the *specific* role of the PVT in these processes, since many of these studies are confounded by the fact that Pavlovian-conditioned reward cues can act not only as “predictors” of reward delivery, but can also come to act as “incentive” stimuli, capable of arousing complex emotional and motivational states (Stewart et al., 1984; Childress et al., 1993; Robinson and Berridge, 1993). It should be noted that here we are referring to incentive stimuli that have Pavlovian conditioned motivational properties, and not instrumental incentive value as described by Dickinson et al. (Balleine and Dickinson, 1998; Dickinson and Balleine, 2002). Pavlovian incentive stimuli have three fundamental properties: (1) they are attractive and elicit approach toward them, as in Pavlovian conditioned approach behavior; (2) they can reinforce the learning of new actions, acting as a conditioned reinforcer; and (3) they can energize ongoing instrumental actions, as in the Pavlovian instrumental transfer (PIT) effect (Estes, 1948; Lovibond, 1983; Berridge, 2001; Cardinal et al., 2002; Holmes et al., 2010). Until recently, it was thought that the conditional relationship between a cue and reward was sufficient to confer incentive motivational value to the cue. That is, if a cue attained predictive value and was capable of eliciting a conditioned response, then it was assumed that it also had the ability to act as an incentive stimulus. However, we have found that this is not the case (Robinson and Flagel, 2009).

**Figure 2:**
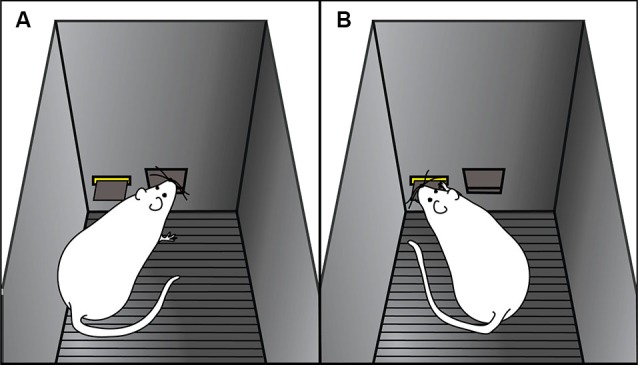
**Cartoon representation of goal-tracking and sign-tracking behaviors.** Examples of **(A)** goal-tracking and **(B)** sign-tracking behaviors in response to lever-cue presentation during a Pavlovian conditioning session. **(A)** Goal-trackers approach the food cup (i.e., location of reward delivery) upon lever-cue presentation. **(B)** Sign-trackers approach the lever-cue during its presentation, even though no response is required for food delivery.

Using an animal model, we have shown that there is considerable variation in the degree to which individuals will attribute predictive and incentive properties to reward-paired cues (Flagel et al., 2007; Robinson and Flagel, 2009; Meyer et al., 2012). When rats are exposed to a classical Pavlovian conditioning paradigm wherein an illuminated lever (conditioned stimulus) is repeatedly paired with delivery of a food reward (unconditioned stimulus), distinct conditioned responses emerge. Some rats, termed goal-trackers, attribute predictive value to the lever-cue, and promptly approach the location of reward delivery upon lever-cue presentation (Figure 2A). Other animals, called sign-trackers, not only attribute predictive value, but also attribute *incentive salience* to the lever-cue, and upon its presentation will approach and manipulate it (Figure 2B), even though no interaction with the lever is required for food delivery. Importantly, all of the animals, regardless of their phenotype, retrieve and eat all of the food pellets, and their behavior during the inter-trial intervals is the same and attenuates over training. Furthermore, if lever presentation is explicitly not paired with food delivery (i.e., unpaired conditions), neither conditioned response develops (Robinson and Flagel, 2009).

There is ample evidence supporting the notion that for sign-trackers, but not goal-trackers, the lever-cue is attributed with incentive salience (e.g., Flagel et al., 2009; Meyer et al., 2012). For sign-trackers the cue itself is attractive and elicits approach—indicative of the first quality of an incentive stimulus (Flagel et al., 2009). Further, for sign-trackers, the lever itself is desirable and acts as a more effective conditioned reinforcer relative to goal-trackers. That is, sign-trackers will respond more than goal-trackers for lever-cue presentation in the absence of food reward (Robinson and Flagel, 2009), demonstrating the second quality of an incentive stimulus. Evidence demonstrating individual variation in the third fundamental property of an incentive stimulus, i.e., general PIT, is lacking, perhaps due to the complex nature of the paradigm. However, there is evidence suggesting that reward cues arouse a conditioned motivational state to a greater extent in sign-trackers than goal-trackers (Saunders and Robinson, 2011, 2012; Saunders et al., 2013a). In sum, the lever-cue is a predictor of reward delivery for both sign- and goal-trackers, as it elicits a conditioned response in both and the responses are learned at the same rate; but only for sign-trackers does the cue serve as an incentive stimulus.

In support of the theory that the attribution of incentive salience to reward cues underlies addiction (Robinson and Berridge, 1993, 2001; Flagel et al., 2009), there is now evidence to suggest that sign-trackers are more likely to exhibit addiction-related behaviors (Saunders et al., 2013b; Robinson et al., 2014). Sign-trackers exhibit a greater propensity for psychomotor sensitization upon repeated treatment with cocaine (Flagel et al., 2008), a form of cocaine-induced plasticity that may contribute to the development of addiction. We have shown that rats who sign-track to food-associated cues do the same for drug-associated cues (Flagel et al., 2010; Yager and Robinson, 2013). Sign-trackers have also been reported to acquire cocaine self-administration more rapidly than goal-trackers (Beckmann et al., 2011). Further, cocaine-associated cues gain inordinate control over drug-taking behavior for sign-trackers, and these animals are more likely to exhibit reinstatement of drug-seeking behavior relative to goal-trackers, even in the face of adverse consequences (Saunders and Robinson, 2010, 2011; Saunders et al., 2013a). Sign-trackers are also more impulsive than goal-trackers, another trait associated with addiction liability in both animal models and humans (Belin et al., 2008; Ersche et al., 2010). Thus, individual differences in the propensity to attribute incentive salience to discrete food-paired cues confer vulnerability to addiction-related behaviors. It should be noted, however, that recent evidence suggests that goal-trackers may be more prone to attributing incentive motivational value to contextual stimuli (see Robinson et al., 2014), especially as they relate to drugs of abuse. These newly emerging findings provide further support for the notion that sign-trackers and goal-trackers process motivationally salient information in quite different ways (Flagel et al., 2011a,b; Robinson et al., 2014); and the PVT may play a central role in the underlying processes as it has previously been implicated the conditioned effects of both discrete and contextual reward-associated cues (e.g., Hamlin et al., 2009; James et al., 2011).

### The neurobiology underlying sign- and goal-tracking behavior

Important findings surrounding the neurobiological mechanisms of cue-motivated behaviors have emerged from the sign-tracker/goal-tracker animal model. Exploiting these individual differences in stimulus-reward learning, we demonstrated that dopamine in the NAc core is necessary for both the learning and expression of a sign-tracking, but not goal-tracking, response (Flagel et al., 2011b; Saunders and Robinson, 2012; Saunders et al., 2013a). Thus, dopamine transmission is critical for the attribution of the incentive, but not necessarily the predictive, properties of reward cues. These findings underscore the fact that sign-tracking and goal-tracking are mediated by distinct neurobiological processes, with the former being dopamine-dependent and the latter dopamine-independent.

**Figure 3:**
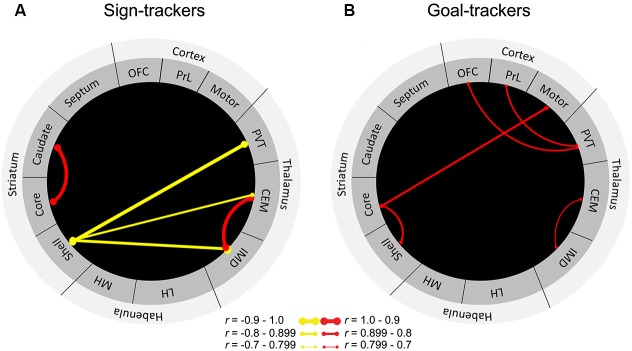
**“Functional connectivity” in sign-trackers and goal-trackers.** Illustration of significantly correlated levels of *c-fos* mRNA expression between brain regions for **(A)** sign-trackers and **(B)** goal-trackers. Red lines are indicative of a significant positive correlation and yellow lines represent negative correlations. The thicker the line, the stronger the correlation. Abbreviations: OFC, orbitofrontal cortex; PrL, prelimbic cortex, PVT, paraventricular nucleus of the thalamus; CEM, centromedial nucleus of the thalamus; IMD, intermediodorsal nucleus of the thalamus; LH, lateral habenula; MH, medial habenula. Adapted from Flagel et al. (2011a).

To further delineate the neural circuitry underlying the attribution of incentive vs. predictive value to reward cues, we sought to examine cue-induced neuronal activity in areas outside of the classic mesocorticolimbic dopamine circuitry. Outbred rats were characterized as sign-trackers vs. goal-trackers based on Pavlovian training sessions consisting of lever-cue presentations paired with food reward. After rats had learned their respective conditioned responses, they were presented with the lever in the absence of food reward to assess cue-induced expression of *c-fos* mRNA throughout the brain. Results showed that levels of *c-fos* mRNA were enhanced in the cortico-striatal-thalamic areas comprising the “motive circuit” (Kelley et al., 2005a) in sign-trackers relative to goal-trackers and controls, who received an equal number of lever-cue and food presentations but in an unpaired fashion (Flagel et al., 2011a). Thus, many parts of the motive circuit are engaged by the *incentive*, and not the predictive, properties of a discrete reward cue. Although sign-trackers exhibited enhanced cue-induced *c-fos* mRNA in all of the midline thalamic nuclei examined (i.e., central medial, intermediodorsal and PVT), the region with the most robust effect was the PVT (Flagel et al., 2011a). In response to cue presentation, sign-trackers exhibited almost twice as much *c-fos* expression in the PVT relative to goal-trackers. Importantly, goal-trackers did not significantly differ from the control group, suggesting that the PVT is highly engaged by cues attributed with incentive, but not predictive value. However, when we examined “functional connectivity” in sign-trackers vs. goal-trackers by identifying correlations in cue-induced *c-fos* mRNA between brain regions, a different picture emerged (Figure 3). Originally, this analysis included only brain regions in which there was a significant difference in cue-induced *c-fos* mRNA between sign-trackers and goal-trackers (Flagel et al., 2011a). Here, however, we have expanded this analysis to include all of the brain areas examined in order to get a more complete picture of network activity in the motive circuit.

In sign-trackers, cue induced *c-fos* mRNA expression was correlated between the thalamus and the NAc shell. Although this correlation was significant for multiple thalamic nuclei, the strongest was a negative correlation (*r* = −0.9) between the PVT and the NAc shell. It should be noted that, with this analysis, the direction of the correlation is uninformative, since the type of cell (e.g., inhibitory or excitatory) in which the *c-fos* is expressed remains unknown. Regardless, this finding further supports a role for the PVT in dopamine-dependent, sub-cortical processing of the sign-tracking response. For goal-trackers, cue-induced *c-fos* mRNA was correlated between the prefrontal cortex and the PVT. Of particular interest is the significant correlation (*r* = 0.7) expressed between the PVT and the PrL, since the densest set of afferents to the PVT comes from the PrL (Li and Kirouac, 2012). Interestingly, there were no significant correlations between the PrL and other thalamic nuclei. There was also evidence of cortico-striatal communication in goal-trackers, which was not present in sign-trackers. These distinct patterns of connectivity highlight the extent to which different neural systems are engaged when a cue is attributed with incentive vs. predictive value and highlight a potential role for the PVT in these learning processes.

### The role of the paraventricular nucleus of the thalamus (PVT) in mediating sign- vs. goal-tracking behaviors

The discovery that PVT activity is increased following exposure to a reward-associated cue in sign-trackers, but not goal-trackers, suggests a *specific* role for the PVT in the attribution of incentive salience. We hypothesize that dopaminergic and orexinergic sub-cortical projections to the PVT, coupled with the dense PVT efferents to the ventral striatum (Li and Kirouac, 2008), may be mediating this process. The mesolimbic dopamine system has long been known to be active in response to reward cues, and the lateral hypothalamus, which contains PVT-projecting orexin neurons, has recently been recognized for a similar role (Choi et al., 2010). It is possible therefore, that exposure to a reward-paired cue elicits robust activity in both dopaminergic and orexinergic projections to the PVT, which could result in increased excitation in PVT neurons. This increased activity in the PVT could ultimately lead to an increase in dopamine activity in the NAc, and may do so to a greater extent than VTA-NAc transmission alone. Presumably, activity in each of these pathways that mediate dopamine release in the NAc are enhanced to a greater extent in sign-trackers than goal-trackers in response to reward cues.

Our previous work has demonstrated that sign-tracking behavior is dependent on dopamine transmission in the NAc core (Flagel et al., 2011b; Saunders and Robinson, 2012); but the role of dopamine in the NAc shell in these behaviors has yet to be investigated. Importantly, the NAc core and shell send direct projections to one another via medium spiny neurons and interneurons (van Dongen et al., 2005). The NAc shell also sends projections directly to the VTA (Nauta et al., 1978; Heimer et al., 1991), and these projections heavily overlap with VTA cells that in turn project back to the NAc core (Haber et al., 2000). Therefore, there are both direct and indirect pathways in which the NAc shell can influence activity in the NAc core. Further, while ample evidence supports a role for the NAc core in cue-reward processing, recent evidence has demonstrated a potentially similar role for the NAc shell (Blaiss and Janak, 2009; Grimm et al., 2011; Peciña and Berridge, 2013). In relation, we found enhanced cue-induced *c-fos* activity in both the core and shell in sign-trackers relative to goal-trackers (Flagel et al., 2011a). Thus, the specific involvement of the NAc core vs. shell in cue-motivated behaviors is not yet entirely clear. We suspect, however, that PVT projections to the NAc affect activity in both the core and the shell and it is, at least in part, via this circuit that the PVT regulates sign-tracking behavior. Based on existing data (Flagel et al., 2011b; Saunders and Robinson, 2012), it is difficult to know whether these effects would occur via modulation of tonic or phasic dopamine release, or both. Nonetheless, this orexin/dopamine-PVT-NAc pathway warrants further investigation as it could play a critical role in incentive salience attribution and prove to be a novel target for the treatment of addiction-related behaviors.

Perhaps more surprising than the discovery of PVT involvement in incentive salience attribution is the new data reported here that PVT and PrL activity is correlated in goal-trackers, but not sign-trackers, following cue presentation. The PrL is important for regulating goal-directed behavior (Balleine and Dickinson, 1998), and has recently been thought to represent a “cognitive-control” mechanism capable of inhibiting conditioned responding to cues (Jonkman et al., 2009; Kober et al., 2010; Mihindou et al., 2013). Indeed, we have shown that goal-trackers exhibit more self-control, as they are found to be less impulsive than sign-trackers (Flagel et al., 2010; Lovic et al., 2011), and perform better on a prefrontal-dependent sustained-attention task (Paolone et al., 2013). Moreover, both the goal-tracking response and cognitively-mediated learning processes are known to be dopamine independent (Dickinson and Balleine, 2002; Flagel et al., 2011b; Saunders and Robinson, 2012; Saunders et al., 2013a). Together, these findings led us to the hypothesis that goal-trackers utilize the discrete reward cue as an informational stimulus which results in the attribution of predictive (but not incentive) value to the cue, via a “top-down” (e.g., PrL-PVT) cognitive learning strategy. In consideration of the circuitry proposed above for sign-trackers, it is possible that for goal-trackers PrL input to the PVT is suppressing the subcortical (i.e., orexinergic/dopaminergic) signaling induced by the reward cue, preventing an increase in accumbens dopamine levels, and thereby preventing the attribution of incentive salience to the cue. For example, the PVT shows dense expression of group II metabotropic glutamate receptors, and agonism of these receptors leads to hyperpolarization of post-synaptic PVT neurons (Hermes and Renaud, 2011). PrL glutamatergic activity at these receptors could therefore result in the suppression of sub-cortical orexin and dopamine signaling at the level of the PVT. Alternatively, PrL input to the PVT could be exciting local GABAergic interneurons, leading to an overall inhibition of the structure, and thereby inhibiting accumbens dopamine activity.

**Figure 4:**
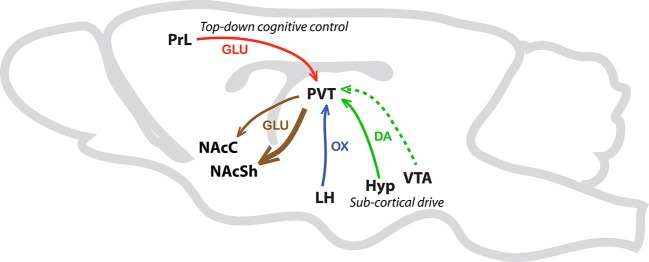
**Schematic illustrating afferents and efferents of interest in the PVT.** This simplified schematic illustrates PVT afferents and efferents that are potentially involved in Pavlovian conditioned approach behavior. The solid green arrow represents sub-cortical dopamine inputs from the hypothalamus (Hyp). The dashed green line represents less dense dopaminergic input from the ventral tegmental area (VTA). The blue arrow represents orexin (OX) input from the lateral hypothalamus (LH) and the red arrow represents glutamatergic (GLU) projections from the prelimbic cortex (PrL) to the PVT. Efferent pathways from the PVT to the nucleus accumbens shell (NAcSh), and to a lesser extent the nucleus accumbens core (NAcC), are represented with brown arrows.

In sum, we are proposing that the PVT is a critical node wherein integration of sub-cortical and cortical inputs can influence the propensity to attribute incentive vs. predictive qualities to discrete reward cues (Figure 4). In support, preliminary data from our lab suggests that lesions of the PVT differentially alter the sign- vs. goal-tracking response (*unpublished data*). Specifically, lesions of the PVT appear to enhance sign-tracking behavior and attenuate goal-tracking behavior. Interestingly, these effects were only apparent in the sign-tracking response after it had been acquired. That is, lesions of the PVT seemed to enhance the vigor of the sign-tracking response, but only during peak performance. In contrast, PVT lesions attenuate both the acquisition and peak performance of the goal-tracking response. It is important to note that these lesions were performed prior to Pavlovian training, and due to the nondescript nature of lesion studies we cannot at this time draw strong conclusions regarding the cell-type or circuitry contributing to the observed effects. Although the proposed mechanisms by which the PVT regulates the attribution of incentive vs. predictive value to reward cues are purely speculative and perhaps oversimplified at this point, our own data and those of others support the notion that the PVT is critical to multiple forms of stimulus-reward learning that are relevant to addiction (Flagel et al., 2009; Robinson et al., 2014).

## Conclusion

Based on anatomical, pharmacological, and behavioral evidence, the PVT appears to play an important role in mediating cue-motivated behaviors. Recent data from our laboratory suggests that the role of the PVT in motivated behavior lies in processing *both* the predictive and incentive properties of reward cues. It is hypothesized that the PVT is a critical regulator in biasing an individual towards either dopamine-dependent (sign-tracking) or dopamine–independent (goal-tracking) behaviors. In this model, sign-tracking behavior is mediated by a “subcortical drive” involving dopaminergic and orexinergic input to the PVT; while “top-down” cognitive control of behavior, in the form of dense glutamatergic PrL innervation of the PVT, underlies goal-tracking behavior. Ongoing studies using optogenetics and Designer Receptors Exclusively Activated by Designer Drugs (DREADD) receptor technology will allow us to further delineate the role of this nucleus and related circuitry in sign- and goal-tracking behaviors. Despite the fact that the PVT has begun to emerge as a major player in motivated behaviors, cue learning, and associated psychopathologies such as addiction, we have only begun to understand this complex nucleus. Further investigations into the function of the PVT, as well as its efferents and afferents, are warranted before we can begin to fully comprehend the neural circuitry underlying motivated behavior.

## Conflict of interest statement

The authors declare that the research was conducted in the absence of any commercial or financial relationships that could be construed as a potential conflict of interest.

## References

[B1] Baik J.-H. (2013). Dopamine signaling in reward-related behaviors. Front. Neural Circuits 7:152 10.3389/fncir.2013.00152 24130517 PMC3795306

[B2] Balleine B. W. Dickinson A. (1998). Goal-directed instrumental action: contingency and incentive learning and their cortical substrates. Neuropharmacology 37, 407–419 10.1016/s0028-3908(98)00033-1 9704982

[B3] Beckmann J. S. Marusich J. A. Gipson C. D. Bardo M. T. (2011). Novelty seeking, incentive salience and acquisition of cocaine self-administration in the rat. Behav. Brain Res. 216, 159–165 10.1016/j.bbr.2010.07.022 20655954 PMC2975769

[B4] Belin D. Mar A. C. Dalley J. W. Robbins T. W. Everitt B. J. (2008). High impulsivity predicts the switch to compulsive cocaine-taking. Science 320, 1352–1355 10.1126/science.1158136 18535246 PMC2478705

[B5] Berridge K. C. (2001). “Reward learning: reinforcement, incentives and expectations,” in Psychology of Learning and Motivation, ed Medin D. (Waltham, MA: Academic Press), 223–278

[B6] Blaiss C. A. Janak P. H. (2009). The nucleus accumbens core and shell are critical for the expression, but not the consolidation, of Pavlovian conditioned approach. Behav. Brain Res. 200, 22–32 10.1016/j.bbr.2008.12.024 19159648 PMC4667776

[B7] Browning J. R. Jansen H. T. Sorg B. A. (2014). Inactivation of the paraventricular thalamus abolishes the expression of cocaine conditioned place preference in rats. Drug Alcohol Depend. 134, 387–390 10.1016/j.drugalcdep.2013.09.021 24139547 PMC3910376

[B8] Cardinal R. N. Parkinson J. A. Lachenal G. Halkerston K. M. Rudarakanchana N. Hall J. (2002). Effects of selective excitotoxic lesions of the nucleus accumbens core, anterior cingulate cortex, and central nucleus of the amygdala on autoshaping performance in rats. Behav. Neurosci. 116, 553–567 10.1037/0735-7044.116.4.553 12148923

[B9] Chen S. Su H.-S. (1990). Afferent connections of the thalamic paraventricular and parataenial nuclei in the rat—a retrograde tracing study with iontophoretic application of Fluoro-Gold. Brain Res. 522, 1–6 10.1016/0006-8993(90)91570-7 2224500

[B10] Childress A. R. Hole A. V. Ehrman R. N. Robbins S. J. Mclellan A. T. O’brien C. P. (1993). Cue reactivity and cue reactivity interventions in drug dependence. NIDA Res. Monogr. 137, 73–95 8289929

[B11] Choi D. L. Davis J. F. Fitzgerald M. E. Benoit S. C. (2010). The role of orexin-A in food motivation, reward-based feeding behavior and food-induced neuronal activation in rats. Neuroscience 167, 11–20 10.1016/j.neuroscience.2010.02.002 20149847

[B12] Choi D. L. Davis J. F. Magrisso I. J. Fitzgerald M. E. Lipton J. W. Benoit S. C. (2012). Orexin signaling in the paraventricular thalamic nucleus modulates mesolimbic dopamine and hedonic feeding in the rat. Neuroscience 210, 243–248 10.1016/j.neuroscience.2012.02.036 22433299 PMC3791334

[B13] Clavier R. Gerfen C. (1982). Intracranial self-stimulation in the thalamus of the rat. Brain Res. Bull. 8, 353–358 10.1016/0361-9230(82)90072-7 7046880

[B14] Cooper R. Taylor L. (1967). Thalamic reticular system and central gray: self stimulation. Science 156, 102–103 10.1126/science.156.3771.102 6020033

[B15] Cornwall J. Phillipson O. T. (1988). Afferent projections to the dorsal thalamus of the rat as shown by retrograde lectin transport. II. The midline nuclei. Brain Res. Bull. 21, 147–161 10.1016/0361-9230(88)90227-4 3191403

[B16] Dayas C. V. Mcgranahan T. M. Martin-Fardon R. Weiss F. (2008). Stimuli linked to ethanol availability activate hypothalamic CART and orexin neurons in a reinstatement model of relapse. Biol. Psychiatry 63, 152–157 10.1016/j.biopsych.2007.02.002 17570346

[B17] Deutch A. Y. Bubser M. Young C. D. (1998). Psychostimulant-induced Fos protein expression in the thalamic paraventricualr nucleus. J. Neurosci. 18, 10680–10687 9852603 10.1523/JNEUROSCI.18-24-10680.1998 PMC6793371

[B18] Deutch A. Y. Ongur D. Duman R. S. (1995). Antipsychotic drugs induce Fos protein in the thalamic paraventricular nucleus: a novel locus of antipsychotic drug action. Neuroscience 66, 337–346 10.1016/0306-4522(94)00571-l 7477876

[B19] Di Ciano P. Benham-Hermetz J. Fogg A. P. Osborne G. E. C. (2007). Role of the prelimbic cortex in the acquisition, re-acquisition or persistence of responding for a drug-paired conditioned reinforcer. Neuroscience 150, 291–298 10.1016/j.neuroscience.2007.09.016 17942235

[B20] Di Pietro N. C. Black Y. D. Kantak K. M. (2006). Context-dependent prefrontal cortex regulation of cocaine self-administration and reinstatement behaviors in rats. Eur. J. Neurosci. 24, 3285–3298 10.1111/j.1460-9568.2006.05193.x 17156389

[B21] Dickinson A. Balleine B. W. (2002). “The role of learning in the operation of motivational systems,” in Stevens’ Book of Experimental Psychology: Learning, Motivation and Emotion, eds Pashler H. Gallistel R. 3rd Edn. (New York: John Wiley and Sons), 497–533

[B22] Ersche K. D. Turton A. J. Pradhan S. Bullmore E. T. Robbins T. W. (2010). Drug addiction endophenotypes: impulsive versus sensation-seeking personality traits. Biol. Psychiatry 68, 770–773 10.1016/j.biopsych.2010.06.015 20678754 PMC3485555

[B23] Estes W. K. (1948). Discriminative conditioning. II. Effects of a Pavlovian conditioned stimulus upon a subsequently established operant response. J. Exp. Psychol. 38, 173–177 10.1037/h0057525 18913666

[B24] Everitt B. J. Robbins T. W. (2013). From the ventral to the dorsal striatum: devolving views of their roles in drug addiction. Neurosci. Biobehav. Rev. 37, 1946–1954 10.1016/j.neubiorev.2013.02.010 23438892

[B25] Flagel S. B. Akil H. Robinson T. E. (2009). Individual differences in the attribution of incentive salience to reward-related cues: implications for addiction. Neuropharmacology 56(Suppl. 1), 139–148 10.1016/j.neuropharm.2008.06.027 18619474 PMC2635343

[B26] Flagel S. B. Cameron C. M. Pickup K. N. Watson S. J. Akil H. Robinson T. E. (2011a). A food predictive cue must be attributed with incentive salience for it to induce c-fos mRNA expression in cortico-striatal-thalamic brain regions. Neuroscience 196, 80–96 10.1016/j.neuroscience.2011.09.004 21945724 PMC3206316

[B27] Flagel S. B. Clark J. J. Robinson T. E. Mayo L. Czuj A. Willuhn I. (2011b). A selective role for dopamine in stimulus-reward learning. Nature 469, 53–57 10.1038/nature09588 21150898 PMC3058375

[B28] Flagel S. B. Robinson T. E. Clark J. J. Clinton S. M. Watson S. J. Seeman P. (2010). An animal model of genetic vulnerability to behavioral disinhibition and responsiveness to reward-related cues: implications for addiction. Neuropsychopharmacology 35, 388–400 10.1038/npp.2009.142 19794408 PMC2794950

[B29] Flagel S. B. Watson S. J. Akil H. Robinson T. E. (2008). Individual differences in the attribution of incentive salience to a reward-related cue: influence on cocaine sensitization. Behav. Brain Res. 186, 48–56 10.1016/j.bbr.2007.07.022 17719099 PMC2225480

[B30] Flagel S. B. Watson S. J. Robinson T. E. Akil H. (2007). Individual differences in the propensity to approach signals vs goals promote different adaptations in the dopamine system of rats. Psychopharmacology (Berl) 191, 599–607 10.1007/s00213-006-0535-8 16972103

[B31] Grace A. A. (2000). The tonic/phasic model of dopamine system regulation and its implications for understanding alcohol and psychostimulant craving. Addiction 95, S119–S128 10.1046/j.1360-0443.95.8s2.1.x 11002907

[B32] García-Cabezas M. Á. Martínez-Sánchez P. Sánchez-González M. Á. Garzón M. Cavada C. (2009). Dopamine innervation in the thalamus: monkey versus rat. Cereb. Cortex 19, 424–434 10.1093/cercor/bhn093 18550594 PMC2638784

[B33] Grimm J. Harkness J. Ratliff C. Barnes J. North K. Collins S. (2011). Effects of systemic or nucleus accumbens-directed dopamine D1 receptor antagonism on sucrose seeking in rats. Psychopharmacology (Berl) 216, 219–233 10.1007/s00213-011-2210-y 21318562 PMC3120924

[B34] Haber S. N. Fudge J. L. Mcfarland N. R. (2000). Striatonigrostriatal pathways in primates form an ascending spiral from the shell to the dorsolateral striatum. J. Neurosci. 20, 2369–2382 10.0270-6474/00/202369-14 10704511 PMC6772499

[B35] Hamlin A. S. Clemens K. J. Choi E. A. Mcnally G. P. (2009). Paraventricular thalamus mediates context-induced reinstatement (renewal) of extinguished reward seeking. Eur. J. Neurosci. 29, 802–812 10.1111/j.1460-9568.2009.06623.x 19200064

[B36] Heimer L. Zahm D. S. Churchill L. Kalivas P. W. Wohltmann C. (1991). Specificity in the projection patterns of accumbal core and shell in the rat. Neuroscience 41, 89–125 10.1016/0306-4522(91)90202-y 2057066

[B37] Hermes M. L. Renaud L. P. (2011). Postsynaptic and presynaptic group II metabotropic glutamate receptor activation reduces neuronal excitability in rat midline paraventricular thalamic nucleus. J. Pharmacol. Exp. Ther. 336, 840–849 10.1124/jpet.110.176149 21139059

[B38] Higley A. E. Kiefer S. W. Li X. Gaál J. Xi Z.-X. Gardner E. L. (2011). Dopamine D3 receptor antagonist SB-277011A inhibits methamphetamine self-administration and methamphetamine-induced reinstatement of drug-seeking in rats. Eur. J. Pharmacol. 659, 187–192 10.1016/j.ejphar.2011.02.046 21466803 PMC3728376

[B39] Holmes N. M. Marchand A. R. Coutureau E. (2010). Pavlovian to instrumental transfer: a neurobehavioural perspective. Neurosci. Biobehav. Rev. 34, 1277–1295 10.1016/j.neubiorev.2010.03.007 20385164

[B40] Hsu D. T. Price J. L. (2009). Paraventricular thalamic nucleus: subcortical connections and innervation by serotonin, orexin and corticotropin-releasing hormone in macaque monkeys. J. Comp. Neurol. 512, 825–848 10.1002/cne.21934 19085970 PMC2646254

[B41] Igelstrom K. M. Herbison A. E. Hyland B. I. (2010). Enhanced c-Fos expression in superior colliculus, paraventricular thalamus and septum during learning of cue-reward association. Neuroscience 168, 706–714 10.1016/j.neuroscience.2010.04.018 20399252

[B42] Ikemoto S. (2010). Brain reward circuitry beyond the mesolimbic dopamine system: a neurobiological theory. Neurosci. Biobehav. Rev. 35, 129–150 10.1016/j.neubiorev.2010.02.001 20149820 PMC2894302

[B43] James M. H. Charnley J. L. Flynn J. R. Smith D. W. Dayas C. V. (2011). Propensity to ‘relapse’ following exposure to cocaine cues is associated with the recruitment of specific thalamic and epithalamic nuclei. Neuroscience 199, 235–242 10.1016/j.neuroscience.2011.09.047 21985936

[B44] James M. H. Charnley J. L. Jones E. Levi E. M. Yeoh J. W. Flynn J. R. (2010). Cocaine- and amphetamine-regulated transcript (CART) signaling within the paraventricular thalamus modulates cocaine-seeking behaviour. PLoS One 5:e12980 10.1371/journal.pone.0012980 20886038 PMC2944892

[B45] James M. H. Dayas C. V. (2013). What about me…? The PVT: a role for the paraventricular thalamus (PVT) in drug-seeking behaviour. Front. Behav. Neurosci. 7:18 10.3389/fnbeh.2013.00018 23509439 PMC3589664

[B46] Johnson Z. V. Revis A. A. Burdick M. A. Rhodes J. S. (2010). A similar pattern of neuronal Fos activation in 10 brain regions following exposure to reward- or aversion-associated contextual cues in mice. Physiol. Behav. 99, 412–418 10.1016/j.physbeh.2009.12.013 20026143 PMC2813902

[B47] Jones M. W. Kilpatrick I. C. Phillipson O. T. (1989). Regulation of dopamine function in the nucleus accumbens of the rat by the thalamic paraventricular nucleus and adjacent midline nuclei. Exp. Brain Res. 76, 572–580 10.1007/bf00248914 2676574

[B48] Jonkman S. Mar A. C. Dickinson A. Robbins T. W. Everitt B. J. (2009). The rat prelimbic cortex mediates inhibitory response control but not the consolidation of instrumental learning. Behav. Neurosci. 123, 875–885 10.1037/a0016330 19634948

[B49] Kelley A. E. Baldo B. A. Pratt W. E. (2005a). A proposed hypothalamic-thalamic-striatal axis for the integration of energy balance, arousal and food reward. J. Comp. Neurol. 493, 72–85 10.1002/cne.20769 16255002

[B50] Kelley A. E. Berridge K. C. (2002). The neuroscience of natural rewards: relevance to addictive drugs. J. Neurosci. 22, 3306–3311 10.0270-6474/02/223306-06 11978804 PMC6758373

[B51] Kelley A. E. Schiltz C. A. Landry C. F. (2005b). Neural systems recruited by drug- and food-related cues: studies of gene activation in corticolimbic regions. Physiol. Behav. 86, 11–14 10.1016/j.physbeh.2005.06.018 16139315

[B52] Khaled M. A. T. M. Farid Araki K. Li B. Coen K. M. Marinelli P. W. Varga J. (2010). The selective dopamine D3 receptor antagonist SB 277011-A, but not the partial agonist BP 897, blocks cue-induced reinstatement of nicotine-seeking. Int. J. Neuropsychopharmacol. 13, 181–190 10.1017/s1461145709991064 19995481

[B53] Kizer J. S. Palkovits M. Brownstein M. J. (1976). The projections of the A8, A9 and A10 dopaminergic cell bodies: evidence for a nigral-hypothalamic-median eminence dopaminergic pathway. Brain Res. 108, 363–370 10.1016/0006-8993(76)90192-x 1276901

[B54] Kober H. Mende-Siedlecki P. Kross E. F. Weber J. Mischel W. Hart C. L. (2010). Prefrontal–striatal pathway underlies cognitive regulation of craving. Proc. Natl. Acad. Sci. U S A 107, 14811–14816 10.1073/pnas.1007779107 20679212 PMC2930456

[B55] Li S. Kirouac G. J. (2008). Projections from the paraventricular nucleus of the thalamus to the forebrain, with special emphasis on the extended amygdala. J. Comp. Neurol. 506, 263–287 10.1002/cne.21502 18022956

[B56] Li S. Kirouac G. J. (2012). Sources of inputs to the anterior and posterior aspects of the paraventricular nucleus of the thalamus. Brain Struct. Funct. 217, 257–273 10.1007/s00429-011-0360-7 22086160

[B57] Lindvall O. Björklund A. Skagerberg G. (1984). Selective histochemical demonstration of dopamine terminal systems in rat di- and telecephalon: new evidence for dopaminergic innervation of hypothalamic neurosecretory nuclei. Brain Res. 306, 19–30 10.1016/0006-8993(84)90352-4 6466973

[B58] Lovibond P. F. (1983). Facilitation of instrumental behavior by a Pavlovian appetitive conditioned stimulus. J. Exp. Psychol. Anim. Behav. Process. 9, 225–247 10.1037/0097-7403.9.3.225 6153052

[B59] Lovic V. Saunders B. T. Yager L. M. Robinson T. E. (2011). Rats prone to attribute incentive salience to reward cues are also prone to impulsive action. Behav. Brain Res. 223, 255–261 10.1016/j.bbr.2011.04.006 21507334 PMC3119757

[B60] Lüscher C. Malenka R. C. (2011). Drug-evoked synaptic plasticity in addiction: from molecular changes to circuit remodeling. Neuron 69, 650–663 10.1016/j.neuron.2011.01.017 21338877 PMC4046255

[B61] Mansour A. Watson S. J. (1995). “Dopamine receptor expression in the central nervous system,” in Psychopharmacology: The Fifth Generation, eds Bloom F. E. Kupfer D. J. (New York: Raven Press, Ltd.), 207–219

[B62] Marchant N. J. Furlong T. M. Mcnally G. P. (2010). Medial dorsal hypothalamus mediates the inhibition of reward seeking after extinction. J. Neurosci. 30, 14102–14115 10.1523/jneurosci.4079-10.2010 20962231 PMC6634760

[B63] Martin-Fardon R. Boutrel B. (2012). Orexin/hypocretin (Orx/Hcrt) transmission and drug-seeking behavior: is the paraventricular nucleus of the thalamus (PVT) part of the drug seeking circuitry? Front. Behav. Neurosci. 6:75 10.3389/fnbeh.2012.00075 23162448 PMC3494007

[B64] Meyer P. J. Lovic V. Saunders B. T. Yager L. M. Flagel S. B. Morrow J. D. (2012). Quantifying individual variation in the propensity to attribute incentive salience to reward cues. PLoS One 7:e38987 10.1371/journal.pone.0038987 22761718 PMC3382216

[B65] Mihindou C. Guillem K. Navailles S. Vouillac C. Ahmed S. H. (2013). Discriminative inhibitory control of cocaine seeking involves the prelimbic prefrontal cortex. Biol. Psychiatry 73, 271–279 10.1016/j.biopsych.2012.08.011 22985696

[B66] Nauta W. J. H. Smith G. P. Faull R. L. M. Domesick V. B. (1978). Efferent connections and nigral afferents of the nucleus accumbens septi in the rat. Neuroscience 3, 385–401 10.1016/0306-4522(78)90041-6 683502

[B67] Nestler E. J. (2014). Epigenetic mechanisms of drug addiction. Neuropharmacology 76(Pt. B), 259–268 10.1016/j.neuropharm.2013.04.004 23643695 PMC3766384

[B68] Paolone G. Angelakos C. C. Meyer P. J. Robinson T. E. Sarter M. (2013). Cholinergic control over attention in rats prone to attribute incentive salience to reward cues. J. Neurosci. 33, 8321–8335 10.1523/jneurosci.0709-13.2013 23658172 PMC3690461

[B150] Parsons M. P. Li S. Kirouac G. J. (2007). Functional and anatomical connection between the paraventricular nucleus of the thalamus and dopamine fibers of the nucleus accumbens. J. Comp. Neurol. 500, 1050–1063 10.1002/cne.21224 17183538

[B69] Paxinos G. Watson C. (2007). The Rat Brain in Stereotaxic Coordinates. Burlingame, MA: Academic Press

[B70] Peciña S. Berridge K. C. (2013). Dopamine or opioid stimulation of nucleus accumbens similarly amplify cue-triggered ‘wanting’ for reward: entire core and medial shell mapped as substrates for PIT enhancement. Eur. J. Neurosci. 37, 1529–1540 10.1111/ejn.12174 23495790 PMC4028374

[B71] Peng X.-Q. Ashby C. R. Jr. Spiller K. Li X. Li J. Thomasson N. (2009). The preferential dopamine D3 receptor antagonist S33138 inhibits cocaine reward and cocaine-triggered relapse to drug-seeking behavior in rats. Neuropharmacology 56, 752–760 10.1016/j.neuropharm.2008.12.007 19136017 PMC3726045

[B72] Pierce R. C. Kalivas P. W. (1997). A circuitry model of the expression of behavioral sensitization to amphetamine-like psychostimulants. Brain Res. Brain Res. Rev. 25, 192–216 10.1016/s0165-0173(97)00021-0 9403138

[B73] Pierce R. C. Reeder D. C. Hicks J. Morgan Z. R. Kalivas P. W. (1997). Ibotenic acid lesions of the dorsal prefrontal cortex disrupt the expression of behavioral sensitization to cocaine. Neuroscience 82, 1103–1114 10.1016/s0306-4522(97)00366-7 9466434

[B74] Pinto A. Jankowski M. Sesack S. R. (2003). Projections from the paraventricular nucleus of the thalamus to the rat prefrontal cortex and nucleus accumbens shell: ultrastructural characteristics and spatial relationships with dopamine afferents. J. Comp. Neurol. 459, 142–155 10.1002/cne.10596 12640666

[B75] Rice O. V. Heidbreder C. A. Gardner E. L. Schonhar C. D. Ashby C. R. (2013). The selective D3 receptor antagonist SB-277011A attenuates morphine-triggered reactivation of expression of cocaine-induced conditioned place preference. Synapse 67, 469–475 10.1002/syn.21653 23404528 PMC4929856

[B76] Robinson T. E. Berridge K. C. (1993). The neural basis of drug craving: an incentive-sensitization theory of addiction. Brain Res. Brain Res. Rev. 18, 247–291 10.1016/0165-0173(93)90013-p 8401595

[B77] Robinson T. E. Berridge K. C. (2001). Incentive-sensitization and addiction. Addiction 96, 103–114 10.1046/j.1360-0443.2001.9611038.x 11177523

[B78] Robinson T. E. Flagel S. B. (2009). Dissociating the predictive and incentive motivational properties of reward-related cues through the study of individual differences. Biol. Psychiatry 65, 869–873 10.1016/j.biopsych.2008.09.006 18930184 PMC2737368

[B79] Robinson T. E. Yager L. M. Cogan E. S. Saunders B. T. (2014). On the motivational properties of reward cues: individual differences. Neuropharmacology 76(Pt. B), 450–459 10.1016/j.neuropharm.2013.05.040 23748094 PMC3796005

[B80] Rocha A. Kalivas P. W. (2010). Role of the prefrontal cortex and nucleus accumbens in reinstating methamphetamine seeking. Eur. J. Neurosci. 31, 903–909 10.1111/j.1460-9568.2010.07134.x 20180839 PMC4346145

[B81] Sánchez-González M. Á. García-Cabezas M. Á. Rico B. Cavada C. (2005). The primate thalamus is a key target for brain dopamine. J. Neurosci. 25, 6076–6083 10.1523/jneurosci.0968-05.2005 15987937 PMC6725054

[B82] Saunders B. T. Robinson T. E. (2010). A cocaine cue acts as an incentive stimulus in some but not others: implications for addiction. Biol. Psychiatry 67, 730–736 10.1016/j.biopsych.2009.11.015 20045508 PMC2849872

[B83] Saunders B. T. Robinson T. E. (2011). Individual variation in the motivational properties of cocaine. Neuropsychopharmacology 36, 1668–1676 10.1038/npp.2011.48 21471956 PMC3138662

[B84] Saunders B. T. Robinson T. E. (2012). The role of dopamine in the accumbens core in the expression of Pavlovian-conditioned responses. Eur. J. Neurosci. 36, 2521–2532 10.1111/j.1460-9568.2012.08217.x 22780554 PMC3424374

[B85] Saunders B. T. Yager L. M. Robinson T. E. (2013a). Cue-evoked cocaine “craving”: role of dopamine in the accumbens core. J. Neurosci. 33, 13989–14000 10.1523/jneurosci.0450-13.2013 23986236 PMC3756749

[B86] Saunders B. T. Yager L. M. Robinson T. E. (2013b). Preclinical studies shed light on individual variation in addiction vulnerability. Neuropsychopharmacology 38, 249–250 10.1038/npp.2012.161 23147491 PMC3521973

[B87] Schiltz C. Bremer Q. Landry C. Kelley A. (2007). Food-associated cues alter forebrain functional connectivity as assessed with immediate early gene and proenkephalin expression. BMC Biol. 5:16 10.1186/1741-7007-5-16 17462082 PMC1868707

[B88] Schiltz C. Kelley A. Landry C. (2005a). Exposure to cues associated with palatable food increases immediate-early gene (IEG) mRNA and proenkephalin (PENK) premRNA expression in the rat brain. Neuropsychopharmacology 30, S213 10.1038/sj.npp.1300970

[B89] Schiltz C. A. Kelley A. E. Landry C. F. (2005b). Contextual cues associated with nicotine administration increase arc mRNA expression in corticolimbic areas of the rat brain. Eur. J. Neurosci. 21, 1703–1711 10.1111/j.1460-9568.2005.04001.x 15845097 PMC1388273

[B90] Stephenson C. P. Hunt G. E. Topple A. N. Mcgregor I. S. (1999). The distribution of 3,4-methylenedioxymethamphetamine “Ecstasy”-induced *c-fos* expression in rat brain. Neuroscience 92, 1011–1023 10.1016/s0306-4522(99)00049-4 10426541

[B91] Stewart J. De Wit H. Eikelboom R. (1984). Role of unconditioned and conditioned drug effects in the self-administration of opiates and stimulants. Psychol. Rev. 91, 251–268 10.1037/0033-295x.91.2.251 6571424

[B92] Su H.-S. Bentivoglio M. (1990). Thalamic midline cell populations projecting to the nucleus accumbens, amygdala and hippocampus in the rat. J. Comp. Neurol. 297, 582–593 10.1002/cne.902970410 1696591

[B93] Takada M. Campbell K. J. Moriizumi T. Hattori T. (1990). On the origin of the dopaminergic innervation of the paraventricular thalamic nucleus. Neurosci. Lett. 115, 33–36 10.1016/0304-3940(90)90513-9 1699175

[B94] Van der Werf Y. D. Witter M. P. Groenewegen H. J. (2002). The intralaminar and midline nuclei of the thalamus. Anatomical and functional evidence for participation in processes of arousal and awareness. Brain Res. Brain Res. Rev. 39, 107–140 10.1016/s0165-0173(02)00181-9 12423763

[B95] van Dongen Y. C. Deniau J. M. Pennartz C. M. A. Galis-De Graaf Y. Voorn P. Thierry A. M. (2005). Anatomical evidence for direct connections between the shell and core subregions of the rat nucleus accumbens. Neuroscience 136, 1049–1071 10.1016/j.neuroscience.2005.08.050 16226842

[B96] Vertes R. P. Hoover W. B. (2008). Projections of the paraventricular and paratenial nuclei of the dorsal midline thalamus in the rat. J. Comp. Neurol. 508, 212–237 10.1002/cne.21679 18311787

[B97] Vogt B. Hof P. Friedman D. Sikes R. Vogt L. (2008). Norepinephrinergic afferents and cytology of the macaque monkey midline, mediodorsal and intralaminar thalamic nuclei. Brain Struct. Funct. 212, 465–479 10.1007/s00429-008-0178-0 18317800 PMC2649766

[B98] Volkow N. D. Wang G.-J. Fischman M. W. Foltin R. W. Fowler J. S. Abumrad N. N. (1997). Relationship between subjective effects of cocaine and dopamine transporter occupancy. Nature 386, 827–830 10.1038/386827a0 9126740

[B99] Wedzony K. Koros E. Czyrak A. Chocyk A. Czepiel K. Fijal K. (2003). Different pattern of brain c-Fos expression following re-exposure to ethanol or sucrose self-administration environment. Naunyn Schmiedebergs Arch. Pharmacol. 368, 331–341 10.1007/s00210-003-0811-7 14574439

[B100] Xi Z.-X. Newman A. H. Gilbert J. G. Pak A. C. Peng X.-Q. Ashby C. R. Jr. (2006). The novel dopamine D3 receptor antagonist NGB 2904 inhibits cocaine’s rewarding effects and cocaine-induced reinstatement of drug-seeking behavior in rats. Neuropsychopharmacology 31, 1393–1405 10.1038/sj.npp.1300912 16205781

[B101] Yager L. M. Robinson T. E. (2013). A classically conditioned cocaine cue acquires greater control over motivated behavior in rats prone to attribute incentive salience to a food cue. Psychopharmacology (Berl) 226, 217–228 10.1007/s00213-012-2890-y 23093382 PMC3570662

[B102] Young C. D. Deutch A. Y. (1998). The effects of thalamic paraventricular nucleus lesions on cocaine-induced locomotor activity and sensitization. Pharmacol. Biochem. Behav. 60, 753–758 10.1016/s0091-3057(98)00051-3 9678661

